# 2-[1-(1-Naphth­yl)-1*H*-1,2,3-triazol-4-yl]pyridine

**DOI:** 10.1107/S160053680901407X

**Published:** 2009-04-30

**Authors:** Bobby Happ, Richard Hoogenboom, Andreas Winter, Martin D. Hager, Stefan O. Baumann, Guido Kickelbick, Ulrich S. Schubert

**Affiliations:** a Laboratory of Organic and Macromolecular Chemistry, Friedrich-Schiller-Universität Jena, Humboldtstrasse 10, 07743 Jena, Germany; b Eindhoven University of Technology, Laboratory of Macromolecular Chemistry and Nanoscience, Den Dolech 2, NL-5612AZ Eindhoven, The Netherlands; c Vienna University of Technology, Institute of Materials Chemistry, Getreidemarkt 9/165, 1060 Wien, Austria

## Abstract

In the crystal structure of the title compound, C_17_H_12_N_4_, the angle between the naphthalene and 1*H*-1,2,3-triazole ring systems is 71.02 (4)° and that between the pyridine and triazole rings is 8.30 (9)°.

## Related literature

For related literature on the synthesis of polypyridyl ligands and 1,2,3-triazole-containing compounds, see: Marin *et al.* (2007 ▶); Winter *et al.* (2007 ▶); Balzani *et al.* (1996 ▶); Newkome *et al.* (2004 ▶); Chan *et al.* (2004 ▶); Rostovtsev *et al.* (2002 ▶); Kolb *et al.* (2001 ▶). The synthesis of the title compound is reported in Happ *et al.* (2009 ▶). For related crystal structures, see: Obata *et al.* (2008 ▶); Schweinfurth *et al.* (2008 ▶); Schulze *et al.* (2009 ▶); Li *et al.* (2007 ▶); Richardson *et al.* (2008 ▶); Angell & Burgess (2007 ▶).
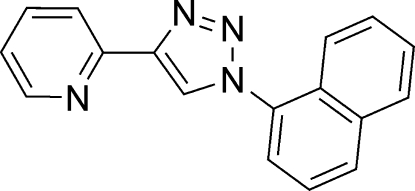


## Experimental

### 

#### Crystal data

C_17_H_12_N_4_
*M* *_r_* = 272.31
Orthorhombic, 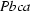

*a* = 11.6378 (4) Å
*b* = 9.3228 (4) Å
*c* = 25.0592 (9) Å
*V* = 2718.84 (18) Å^3^
*Z* = 8
Mo *K*α radiation
μ = 0.08 mm^−1^
*T* = 296 K
0.63 × 0.18 × 0.07 mm

#### Data collection

Bruker Kappa APEXII diffractometer
Absorption correction: multi-scan (*SADABS*; Sheldrick, 2008*a* ▶) *T*_min_ = 0.950, *T*_max_ = 0.994
14517 measured reflections
2391 independent reflections
1934 reflections with *I* > 2σ(*I*)
*R*_int_ = 0.027

#### Refinement

*R*[*F* ^2^ > 2σ(*F* ^2^)] = 0.035
*wR*(*F* ^2^) = 0.120
*S* = 1.01
2391 reflections
190 parameters
H-atom parameters constrained
Δρ_max_ = 0.14 e Å^−3^
Δρ_min_ = −0.18 e Å^−3^

### 

Data collection: *APEX2* (Bruker, 2008 ▶); cell refinement: *SAINT* (Bruker, 2008 ▶); data reduction: *SAINT*; program(s) used to solve structure: *SHELXS97* (Sheldrick, 2008*b* ▶); program(s) used to refine structure: *SHELXL97* (Sheldrick, 2008*b* ▶); molecular graphics: *CARINE* (Boudias & Monceau, 1998 ▶); software used to prepare material for publication: *SHELXTL* (Sheldrick, 2008*b* ▶).

## Supplementary Material

Crystal structure: contains datablocks I, global. DOI: 10.1107/S160053680901407X/wn2322sup1.cif

Structure factors: contains datablocks I. DOI: 10.1107/S160053680901407X/wn2322Isup2.hkl

Additional supplementary materials:  crystallographic information; 3D view; checkCIF report

## Figures and Tables

**Fig. 1.**
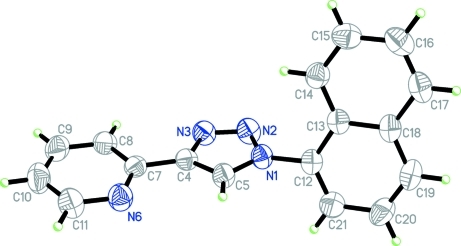
The molecular structure of the title compound. Displacement ellipsoids are drawn at the 50% probability level and H atoms are shown as spheres of arbitrary radii.

**Fig. 2.**
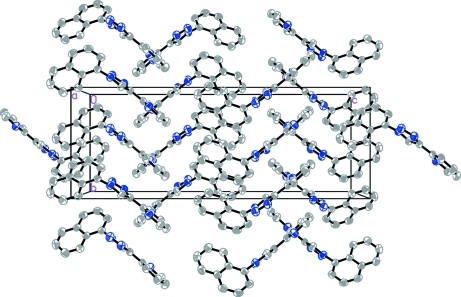
A plot of the molecular packing of the title compound. Displacement ellipsoids are drawn at the 50% probability level. H atoms have been omitted for clarity.

## supplementary crystallographic information

### Comment

The development of novel functional materials for applications in solar cells or LEDs represents a major challenge in current materials science. Transition metal complexes have been investigated to extend the application possibilities in modern device technology (Marin *et al.*, 2007; Ulbricht *et al.*, 2009). Ru^II^ complexes of bipyridine-type ligands are highly interesting due to their predictable electro-optical properties (Balzani *et al.*, 1996). The syntheses of functionalized 2,2'-bipyridines have been reviewed, revealing that the selective and easy synthesis of mono-functionalized ligands remains a synthetic challenge (Marin *et al.*, 2007; Newkome *et al.*, 2004). Li *et al.* (2007) and Obata *et al.* (2008) showed that the 1*H*-1,2,3-triazole ring system can serve as an alternative for pyridine in oligopyridine ligands. As comparable structures to 2,2':6',2''-terpyridines these examples have demonstrated the versatility of this approach to substitute pyridine rings of the oligopyridine ligands by functionalized triazoles (Schulze *et al.*, 2009; Li *et al.*, 2007). In order to explore the properties of such functionalized bidentate ligands, we have synthesized a library of pyridin-2-yl substituted 1*H*-1,2,3-triazole systems (Happ *et al.*, 2009), utlizing the so-called Click reaction (Chan *et al.*, 2004; Rostovtsev *et al.*, 2002; Kolb *et al.*, 2001) and their Ru^II^ complexes.

The crystal structures of 1-substituted 2-(1*H*-1,2,3-triazol-4-yl)pyridines have thus far been rarely discussed in the literature. The crystal structures of derivatives bearing a 4'-butyloxybenzene (Schweinfurth *et al.*, 2008) or benzyl group (Obata *et al.*, 2008) in the 1-position of the 1*H*-1,2,3-triazole ring have been reported. The structure of an unsubstituted derivative was studied by Richardson *et al.* (2008). Furthermore, the crystal structure of a dimeric species was discussed by Angell & Burgess (2007). The crystal structures of metal complexes of 2-(1*H*-1,2,3-triazol-4-yl)pyridines have been more extensively investigated. The structures of various Ru^II^ (Schulze *et al.*, 2009; Li *et al.*, 2007), Fe^II^ (Li *et al.*, 2007) and Re^I^ complexes (Obata *et al.*, 2008) were reported recently.

Here we report the crystal structure of the title compound. The geometric parameters are in good agreement with literature values (Schweinfurth *et al.*, 2008). The pyridine ring and the triazole ring are nearly coplanar and the N atoms N3 and N6 show the expected *anti* configuration. The planes through these two heterocyclic ring systems (N1–C5 and N6–C11) deviate only by an angle of 8.30 (9)°. The naphthalene (C12–C21) and triazole (N1–C5) ring systems are inclined at an angle of 71.02 (4)°.

### Experimental

The title compound, C_17_H_12_N_4_, was synthesized as reported previously (Happ *et al.*, 2009): Sodium azide (6 mmol) and anhydrous CuSO_4_ (62 mg, 0.4 mmol) were dissolved in dry methanol (15 ml) in a 20 ml microwave vial. (Naphthalen-1-yl)boronic acid (663 mg, 3.84 mmol) was added to the brown solution and the mixture was stirred for 17 h at room temperature. The progress of the reaction was monitored by TLC (SiO_2_, CHCl_3_ as eluent). Then CuSO_4_.5H_2_O (30 mg, 0.2 mmol), sodium ascorbate (384 mg, 1.95 mmol), 2-ethynylpyridine (435 mg, 4.2 mmol) and water (5 ml) were added. The reaction mixture was heated under microwave irradiation at 100 °C for 1 h. Water (30 ml) was added and the product was extracted with toluene (3 × 15 ml). After drying (MgSO_4_) and evaporation of the solvent, the crude product was purified by column chromatography [Al_2_O_3_, CH_2_Cl_2_/EtOAc (1:1 ratio) as eluent]. The title compound was isolated as a white crystalline solid (673 mg, 64%). Single crystals of the purified compound were obtained by slow evaporation of a CH_2_Cl_2_/*n*-hexane solution (2:1 ratio).

### Refinement

H atoms were placed in idealized positions with C—H = 0.93 Å and refined as riding on their parent atoms with *U*_iso_(H) = 1.2*U*_eq_(C).

### Crystal data

**Table:**

C_17_H_12_N_4_	*D*_x_ = 1.330 Mg m^−^^3^
*M**_r_* = 272.31	Mo *K*α radiation, λ = 0.71073 Å
Orthorhombic, *P**b**c**a*	Cell parameters from 4456 reflections
*a* = 11.6378 (4) Å	θ = 2.4–20.9°
*b* = 9.3228 (4) Å	µ = 0.08 mm^−^^1^
*c* = 25.0592 (9) Å	*T* = 296 K
*V* = 2718.84 (18) Å^3^	Stick, colourless
*Z* = 8	0.63 × 0.18 × 0.07 mm
*F*(000) = 1136	

### Data collection

**Table:**

Bruker Kappa APEXII diffractometer	2391 independent reflections
Radiation source: fine-focus sealed tube	1934 reflections with *I* > 2σ(*I*)
graphite	*R*_int_ = 0.027
ω scans	θ_max_ = 25.0°, θ_min_ = 1.6°
Absorption correction: multi-scan (*SADABS*; Sheldrick, 2008a)	*h* = −13→13
*T*_min_ = 0.950, *T*_max_ = 0.994	*k* = −10→11
14517 measured reflections	*l* = −28→29

### Refinement

**Table:**

Refinement on *F*^2^	Primary atom site location: structure-invariant direct methods
Least-squares matrix: full	Secondary atom site location: difference Fourier map
*R*[*F*^2^ > 2σ(*F*^2^)] = 0.035	Hydrogen site location: inferred from neighbouring sites
*wR*(*F*^2^) = 0.120	H-atom parameters constrained
*S* = 1.01	*w* = 1/[σ^2^(*F*_o_^2^) + (0.0842*P*)^2^ + 0.1848*P*] where *P* = (*F*_o_^2^ + 2*F*_c_^2^)/3
2391 reflections	(Δ/σ)_max_ < 0.001
190 parameters	Δρ_max_ = 0.14 e Å^−^^3^
0 restraints	Δρ_min_ = −0.18 e Å^−^^3^

### Special details

**Table:**

Geometry. All e.s.d.'s (except the e.s.d. in the dihedral angle between two l.s. planes) are estimated using the full covariance matrix. The cell e.s.d.'s are taken into account individually in the estimation of e.s.d.'s in distances, angles and torsion angles; correlations between e.s.d.'s in cell parameters are only used when they are defined by crystal symmetry. An approximate (isotropic) treatment of cell e.s.d.'s is used for estimating e.s.d.'s involving l.s. planes.
Refinement. Refinement of *F*^2^ against ALL reflections. The weighted *R*-factor *wR* and goodness of fit *S* are based on *F*^2^, conventional *R*-factors *R* are based on *F*, with *F* set to zero for negative *F*^2^. The threshold expression of *F*^2^ > σ(*F*^2^) is used only for calculating *R*-factors(gt) *etc*. and is not relevant to the choice of reflections for refinement. *R*-factors based on *F*^2^ are statistically about twice as large as those based on *F*, and *R*- factors based on ALL data will be even larger.

### Fractional atomic coordinates and isotropic or equivalent isotropic displacement parameters (Å^2^)

**Table:**

	*x*	*y*	*z*	*U*_iso_*/*U*_eq_	
N1	0.20019 (9)	0.39250 (12)	0.37942 (4)	0.0428 (3)	
N2	0.31490 (10)	0.36728 (14)	0.37933 (5)	0.0509 (3)	
N3	0.35790 (10)	0.43998 (13)	0.33925 (5)	0.0489 (3)	
C4	0.27219 (11)	0.51071 (14)	0.31370 (5)	0.0403 (3)	
C5	0.17170 (12)	0.48053 (15)	0.33923 (5)	0.0450 (4)	
H5	0.0987	0.5139	0.3306	0.054*	
N6	0.19978 (11)	0.66666 (14)	0.24668 (5)	0.0530 (4)	
C7	0.29352 (12)	0.60832 (15)	0.26887 (5)	0.0411 (3)	
C8	0.40394 (13)	0.64153 (17)	0.25220 (5)	0.0495 (4)	
H8	0.4672	0.5971	0.2678	0.059*	
C9	0.41850 (15)	0.74114 (18)	0.21229 (6)	0.0590 (4)	
H9	0.4918	0.7662	0.2008	0.071*	
C10	0.32349 (16)	0.80283 (19)	0.18976 (7)	0.0635 (5)	
H10	0.3310	0.8711	0.1629	0.076*	
C11	0.21677 (15)	0.76197 (19)	0.20758 (7)	0.0619 (5)	
H11	0.1526	0.8030	0.1915	0.074*	
C12	0.12717 (11)	0.32768 (14)	0.41875 (5)	0.0420 (3)	
C13	0.13387 (11)	0.37575 (14)	0.47248 (5)	0.0405 (3)	
C14	0.20520 (12)	0.48862 (16)	0.48997 (6)	0.0482 (4)	
H14	0.2525	0.5357	0.4657	0.058*	
C15	0.20518 (14)	0.52911 (18)	0.54222 (7)	0.0573 (4)	
H15	0.2519	0.6044	0.5532	0.069*	
C16	0.13572 (14)	0.4588 (2)	0.57950 (6)	0.0607 (4)	
H16	0.1381	0.4860	0.6152	0.073*	
C17	0.06506 (14)	0.35138 (18)	0.56387 (6)	0.0552 (4)	
H17	0.0187	0.3060	0.5890	0.066*	
C18	0.06071 (12)	0.30717 (15)	0.50993 (5)	0.0445 (4)	
C19	−0.01489 (14)	0.19830 (16)	0.49262 (7)	0.0554 (4)	
H19	−0.0625	0.1532	0.5173	0.066*	
C20	−0.01924 (15)	0.15834 (17)	0.44059 (7)	0.0587 (4)	
H20	−0.0703	0.0872	0.4298	0.070*	
C21	0.05301 (13)	0.22392 (16)	0.40296 (6)	0.0523 (4)	
H21	0.0500	0.1963	0.3673	0.063*	

### Atomic displacement parameters (Å^2^)

**Table:**

	*U*^11^	*U*^22^	*U*^33^	*U*^12^	*U*^13^	*U*^23^
N1	0.0377 (6)	0.0483 (6)	0.0423 (6)	0.0049 (5)	0.0060 (5)	0.0034 (5)
N2	0.0405 (7)	0.0621 (8)	0.0501 (7)	0.0109 (6)	0.0055 (5)	0.0043 (6)
N3	0.0401 (7)	0.0593 (8)	0.0472 (7)	0.0042 (6)	0.0065 (5)	−0.0002 (6)
C4	0.0378 (7)	0.0452 (7)	0.0378 (7)	0.0007 (6)	0.0040 (5)	−0.0052 (6)
C5	0.0374 (7)	0.0524 (8)	0.0451 (8)	0.0036 (6)	0.0028 (6)	0.0066 (6)
N6	0.0468 (8)	0.0585 (8)	0.0536 (8)	0.0012 (6)	0.0035 (5)	0.0076 (6)
C7	0.0415 (8)	0.0447 (7)	0.0371 (7)	−0.0040 (6)	0.0043 (5)	−0.0063 (6)
C8	0.0425 (9)	0.0630 (9)	0.0429 (8)	−0.0055 (7)	0.0058 (6)	−0.0039 (6)
C9	0.0571 (10)	0.0706 (10)	0.0493 (9)	−0.0173 (8)	0.0161 (7)	−0.0035 (8)
C10	0.0792 (12)	0.0613 (10)	0.0500 (9)	−0.0061 (9)	0.0136 (8)	0.0087 (8)
C11	0.0632 (11)	0.0660 (10)	0.0566 (10)	0.0047 (8)	0.0022 (8)	0.0104 (8)
C12	0.0403 (7)	0.0404 (7)	0.0454 (8)	0.0062 (6)	0.0061 (6)	0.0045 (6)
C13	0.0383 (8)	0.0397 (7)	0.0437 (7)	0.0086 (6)	0.0013 (5)	0.0054 (6)
C14	0.0421 (8)	0.0502 (8)	0.0524 (9)	0.0010 (6)	0.0019 (6)	0.0040 (7)
C15	0.0517 (9)	0.0619 (10)	0.0581 (9)	0.0027 (7)	−0.0055 (7)	−0.0072 (8)
C16	0.0589 (10)	0.0774 (11)	0.0459 (9)	0.0123 (9)	−0.0010 (7)	−0.0057 (8)
C17	0.0543 (9)	0.0658 (10)	0.0455 (8)	0.0103 (8)	0.0089 (7)	0.0093 (7)
C18	0.0427 (8)	0.0440 (8)	0.0469 (8)	0.0088 (6)	0.0070 (6)	0.0089 (6)
C19	0.0540 (9)	0.0483 (8)	0.0639 (10)	−0.0024 (7)	0.0152 (7)	0.0106 (7)
C20	0.0588 (10)	0.0460 (8)	0.0713 (11)	−0.0111 (7)	0.0087 (8)	−0.0034 (8)
C21	0.0562 (9)	0.0477 (9)	0.0529 (9)	0.0017 (7)	0.0047 (7)	−0.0053 (6)

### Geometric parameters (Å, °)

**Table:**

N1—C5	1.3408 (17)	C12—C21	1.355 (2)
N1—N2	1.3556 (16)	C12—C13	1.4212 (19)
N1—C12	1.4348 (17)	C13—C14	1.410 (2)
N2—N3	1.3110 (16)	C13—C18	1.4195 (19)
N3—C4	1.3563 (18)	C14—C15	1.363 (2)
C4—C5	1.3623 (19)	C14—H14	0.9300
C4—C7	1.4669 (19)	C15—C16	1.398 (2)
C5—H5	0.9300	C15—H15	0.9300
N6—C11	1.337 (2)	C16—C17	1.354 (2)
N6—C7	1.3398 (19)	C16—H16	0.9300
C7—C8	1.386 (2)	C17—C18	1.414 (2)
C8—C9	1.375 (2)	C17—H17	0.9300
C8—H8	0.9300	C18—C19	1.411 (2)
C9—C10	1.368 (2)	C19—C20	1.357 (2)
C9—H9	0.9300	C19—H19	0.9300
C10—C11	1.374 (2)	C20—C21	1.403 (2)
C10—H10	0.9300	C20—H20	0.9300
C11—H11	0.9300	C21—H21	0.9300
			
C5—N1—N2	110.40 (11)	C13—C12—N1	119.05 (12)
C5—N1—C12	128.86 (11)	C14—C13—C12	124.20 (12)
N2—N1—C12	120.75 (11)	C14—C13—C18	118.92 (13)
N3—N2—N1	106.70 (11)	C12—C13—C18	116.84 (13)
N2—N3—C4	109.41 (11)	C15—C14—C13	120.35 (14)
N3—C4—C5	108.01 (12)	C15—C14—H14	119.8
N3—C4—C7	122.59 (12)	C13—C14—H14	119.8
C5—C4—C7	129.27 (12)	C14—C15—C16	120.81 (16)
N1—C5—C4	105.48 (12)	C14—C15—H15	119.6
N1—C5—H5	127.3	C16—C15—H15	119.6
C4—C5—H5	127.3	C17—C16—C15	120.30 (15)
C11—N6—C7	116.96 (13)	C17—C16—H16	119.9
N6—C7—C8	122.62 (13)	C15—C16—H16	119.9
N6—C7—C4	115.59 (12)	C16—C17—C18	120.93 (14)
C8—C7—C4	121.75 (13)	C16—C17—H17	119.5
C9—C8—C7	118.96 (15)	C18—C17—H17	119.5
C9—C8—H8	120.5	C19—C18—C17	121.72 (13)
C7—C8—H8	120.5	C19—C18—C13	119.63 (13)
C10—C9—C8	118.97 (15)	C17—C18—C13	118.64 (14)
C10—C9—H9	120.5	C20—C19—C18	121.07 (14)
C8—C9—H9	120.5	C20—C19—H19	119.5
C9—C10—C11	118.68 (16)	C18—C19—H19	119.5
C9—C10—H10	120.7	C19—C20—C21	120.23 (15)
C11—C10—H10	120.7	C19—C20—H20	119.9
N6—C11—C10	123.78 (16)	C21—C20—H20	119.9
N6—C11—H11	118.1	C12—C21—C20	119.76 (14)
C10—C11—H11	118.1	C12—C21—H21	120.1
C21—C12—C13	122.44 (13)	C20—C21—H21	120.1
C21—C12—N1	118.51 (13)		
			
C5—N1—N2—N3	0.24 (15)	C5—N1—C12—C13	−109.53 (16)
C12—N1—N2—N3	179.90 (12)	N2—N1—C12—C13	70.88 (16)
N1—N2—N3—C4	−0.21 (15)	C21—C12—C13—C14	−176.38 (14)
N2—N3—C4—C5	0.12 (16)	N1—C12—C13—C14	2.65 (19)
N2—N3—C4—C7	176.41 (12)	C21—C12—C13—C18	1.60 (19)
N2—N1—C5—C4	−0.16 (15)	N1—C12—C13—C18	−179.37 (12)
C12—N1—C5—C4	−179.79 (13)	C12—C13—C14—C15	179.13 (13)
N3—C4—C5—N1	0.03 (16)	C18—C13—C14—C15	1.2 (2)
C7—C4—C5—N1	−175.93 (13)	C13—C14—C15—C16	0.7 (2)
C11—N6—C7—C8	−1.1 (2)	C14—C15—C16—C17	−1.7 (2)
C11—N6—C7—C4	176.54 (13)	C15—C16—C17—C18	0.6 (2)
N3—C4—C7—N6	177.95 (12)	C16—C17—C18—C19	−178.08 (14)
C5—C4—C7—N6	−6.6 (2)	C16—C17—C18—C13	1.3 (2)
N3—C4—C7—C8	−4.4 (2)	C14—C13—C18—C19	177.22 (13)
C5—C4—C7—C8	171.04 (14)	C12—C13—C18—C19	−0.87 (18)
N6—C7—C8—C9	1.8 (2)	C14—C13—C18—C17	−2.18 (19)
C4—C7—C8—C9	−175.67 (13)	C12—C13—C18—C17	179.73 (12)
C7—C8—C9—C10	−0.9 (2)	C17—C18—C19—C20	179.09 (14)
C8—C9—C10—C11	−0.5 (3)	C13—C18—C19—C20	−0.3 (2)
C7—N6—C11—C10	−0.5 (3)	C18—C19—C20—C21	0.8 (2)
C9—C10—C11—N6	1.3 (3)	C13—C12—C21—C20	−1.1 (2)
C5—N1—C12—C21	69.54 (19)	N1—C12—C21—C20	179.83 (13)
N2—N1—C12—C21	−110.05 (15)	C19—C20—C21—C12	−0.1 (2)
